# The contribution of musculoskeletal factors to physical frailty: a cross-sectional study

**DOI:** 10.1186/s12891-021-04795-4

**Published:** 2021-11-01

**Authors:** Monica C. Tembo, Mohammadreza Mohebbi, Kara L. Holloway-Kew, James Gaston, Sophia X. Sui, Sharon L. Brennan-Olsen, Lana J. Williams, Mark A. Kotowicz, Julie A. Pasco

**Affiliations:** 1 Epi-Centre for Healthy Ageing, IMPACT – The Institute for Mental and Physical Health and Clinical Translation, School of Medicine, Barwon Health, Deakin University, PO Box 281, Geelong, VIC 3220 Australia; 2 Faculty of Health, Biostatistics Unit, Deakin University, Geelong, VIC Australia; 3 School of Health and Social Development, Deakin University, Waterfront Geelong Campus, Geelong, VIC Australia; 4 Institute for Health Transformation, Deakin University, Waterfront Geelong Campus, Geelong, VIC Australia; 5 Department of Medicine-Western Health, The University of Melbourne, St Albans, VIC Australia; 6 Australian Institute for Musculoskeletal Science (AIMSS), The University of Melbourne, St Albans, VIC Australia; 7 Barwon Health, Geelong, VIC Australia; 8 Department of Epidemiology and Preventive Medicine, Monash University, Melbourne, VIC Australia

**Keywords:** Ageing, Frailty, Lean mass, Osteoporosis, Osteosarcopenia, Physical performance, Sarcopenia

## Abstract

**Background:**

Musculoskeletal conditions and physical frailty have overlapping constructs. We aimed to quantify individual contributions of musculoskeletal factors to frailty.

**Methods:**

Participants included 347 men and 360 women aged ≥60 yr (median ages; 70.8 (66.1–78.6) and 71.0 (65.2–77.5), respectively) from the Geelong Osteoporosis Study. Frailty was defined as ≥3, pre-frail 1–2, and robust 0, of the following; unintentional weight loss, weakness, low physical activity, exhaustion, and slowness. Measures were made of femoral neck BMD, appendicular lean mass index (ALMI, kg/m^2^) and whole-body fat mass index (FMI, kg/m^2^) by DXA (Lunar), SOS, BUA and SI at the calcaneus (Lunar Achilles Insight) and handgrip strength by dynamometers. Binary and ordinal logistic regression models and AUROC curves were used to quantify the contribution of musculoskeletal parameters to frailty. Potential confounders included anthropometry, smoking, alcohol, prior fracture, FMI, SES and comorbidities.

**Results:**

Overall, 54(15.6%) men and 62(17.2%) women were frail. In adjusted-binary logistic models, SI, ALMI and HGS were associated with frailty in men (OR = 0.73, 95%CI 0.53–1.01; OR=0.48, 0.34–0.68; and OR = 0.11, 0.06–0.22; respectively). Muscle measures (ALMI and HGS) contributed more to this association than did bone (SI) (AUROCs 0.77, 0.85 vs 0.71, respectively). In women, only HGS was associated with frailty in adjusted models (OR = 0.30 95%CI 0.20–0.45, AUROC = 0.83). In adjusted ordinal models, similar results were observed in men; for women, HGS and ALMI were associated with frailty (ordered OR = 0.30 95%CI 0.20–0.45; OR = 0.56, 0.40–0.80, respectively).

**Conclusion:**

Muscle deficits appeared to contribute more than bone deficits to frailty. This may have implications for identifying potential musculoskeletal targets for preventing or managing the progression of frailty.

**Supplementary Information:**

The online version contains supplementary material available at 10.1186/s12891-021-04795-4.

## Background

With the progressive age, the prevalence of frailty increases [1–3]. Frailty, a clinical syndrome that affects multiple physiological systems, is associated with a diminished functional reserve and an increased vulnerability to adverse events such as falls and fractures [4], as well as minor stressors such as colds [5, 6]. There are a variety of definitions for frailty depending on the assessment tool used [5]. However, the two models that dominate the literature and are validated in large populations are the Fried frailty phenotype and the frailty index of accumulation of deficits [6, 7]. The former focuses on the physical phenotype of frailty and considers the deterioration of physical performance and robustness. In contrast, the frailty index of deficit accumulation includes biomedical and psychosocial factors to identify frailty on a cumulative scale [7].

With ageing, there is a reduction of bone mass, muscle mass and strength, which may result in osteoporosis, sarcopenia or a combination of the two, known as osteosarcopenia [8–10]. Research has shown that musculoskeletal conditions are a major cause of functional impairment and disability [11, 12] and that frailty is associated with lower bone mineral density (BMD), muscle or lean mass and handgrip strength (HGS) [6, 13–15]. However, to our knowledge, to date, no study has quantified the contribution of musculoskeletal components to frailty. This is pertinent to the understanding the condition and highlighting potential targets for interventions. Thus, the aim of this study was to investigate the association between musculoskeletal factors and frailty, defined using the Fried frailty phenotype, and quantify their contributions to frailty.

## Methods

**Fig. 1:**
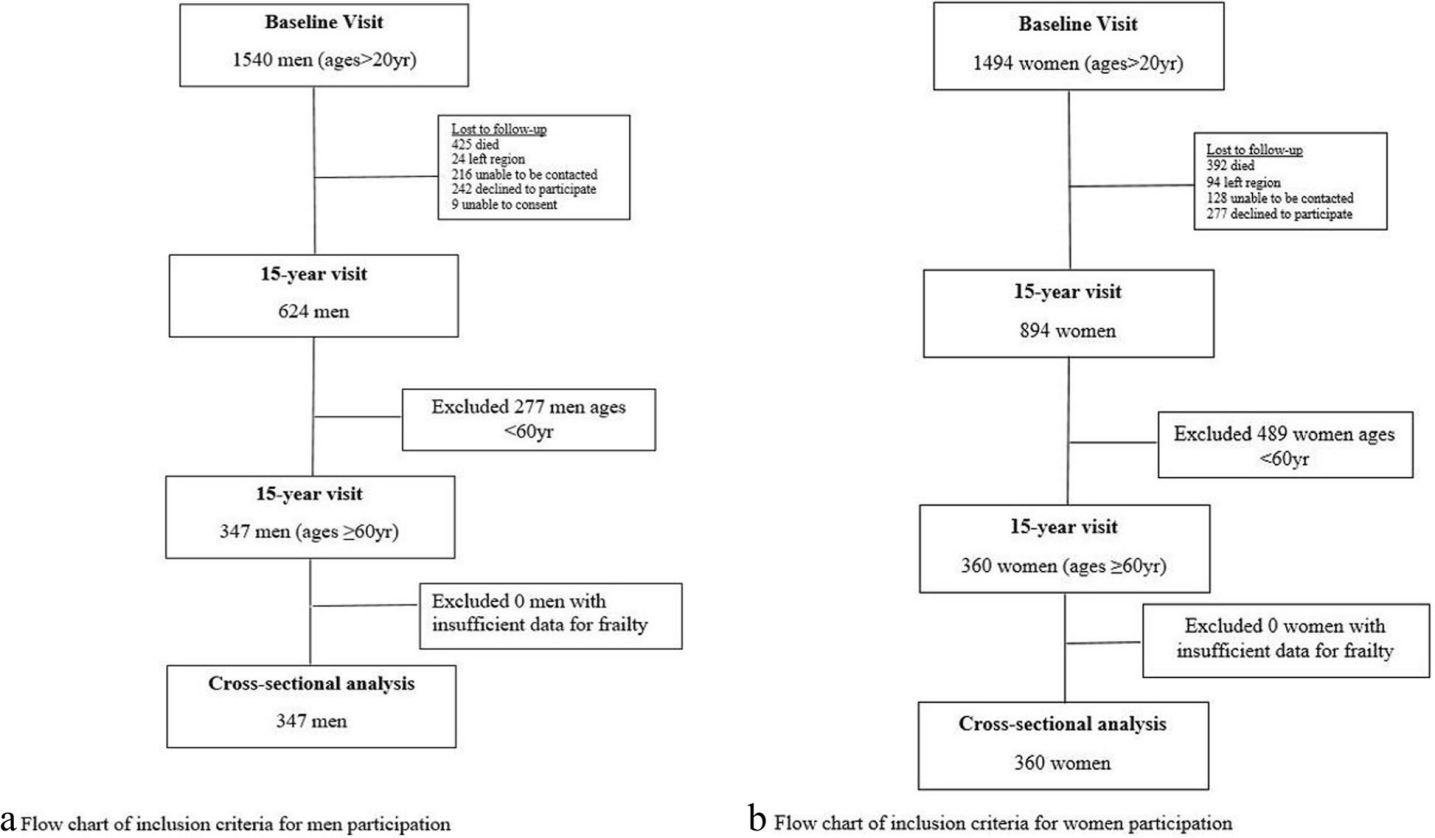
Flow charts of inclusion criteria for participation

This cross-sectional study included men and women enrolled in Geelong Osteoporosis Study (GOS). GOS is an ongoing population-based cohort study involving more than 3200 randomly selected adults (approximately 98% Caucasian) from the Barwon Statistical Division in south-eastern Australia. Full details of the study have been published elsewhere [16]. For these analyses, we utilised cross-sectional data from the 15-yr follow-up assessment phase for both men (2016–2019) and women (2011–2014). Men and women aged ≥60 yr who provided sufficient data for addressing Fried frailty criteria were included in this study (*n* = 360 and *n* = 347 respectively) (Fig. 1a and b). There were no exclusions for comorbidities or behaviours.

The study was approved by the Human Research Ethics Committee at Barwon Health. All procedures performed in studies involving human participants were in accordance with the ethical standards of the institutional and national research committees and with the 1964 Helsinki declaration and its later amendments or comparable ethical standards.

Written informed consent was obtained from all participants in the study.

### Visit assessment

Participants completed questionnaires that documented physical activity [17] dietary information [18], lifestyle factors and health behaviours, sociodemographic factors, medications, comorbidities and post-baseline fractures that were radiologically confirmed.

### Frailty assessment

Frailty was identified using a modified Fried frailty phenotype [6], which categorised individuals into frail, pre-frail or robust groups, based on the responses to five criteria including unintentional weight loss, weakness, low physical activity, exhaustion and slowness. Weakness was determined using handgrip strength (HGS) measured with a hand-held Jamar dynamometer (Sammons Preston, Bolingbrook, IL, UK) for women [19] and Vernier dynamometer (Venier Software and Technology, Beaverton, USA) for men. Vernier values were converted to Jamar equivalents using an equation previously described [20]. HGS values below cut-points equivalent to the lowest 20% stratified by sex and body mass index (BMI) were considered as weakness [6]. The HGS procedure was demonstrated to participants before the trials. For women, there was no interval between trials, and for men, there was a 3 s interval between the trials; for both sexes the mean of the maximum reading from each hand was used in analyses. Slowness was measured using the Timed Up & Go (TUG) test that measures functional mobility including balance and muscle performance [21]. The time was recorded for the participant to stand from a chair (standard height without armrests), walk 3 m, turn around and return to sit back in the chair. A score ≥ 10s was considered as slow [22]. Unintentional weight loss, exhaustion and low physical activity were self-reported. Low physical activity was ascertained using a single question that asked about the current mobility status of participations. Low physical activity was indicated when participants selected one of the following responses: “limited”, “inactive”, “chair” or “bedridden”, or “bedfast”. Participants with at least three of the five items in the modified Fried tool were categorised as frail, 1–2 items as pre-frail, and zero items as robust.

### Musculoskeletal assessment

Using electronic scales and a wall-mounted Harpenden stadiometer, weight and height were measured to the nearest 0.1 kg and 0.1 cm, respectively, and BMI was calculated (kg/m^2^). Using dual-energy X-ray absorptiometry (DXA, Lunar Prodigy Pro), areal bone mineral density (BMD) was measured at the femoral neck, and measures of total and regional body fat and lean tissue mass were derived from the whole-body scans. DXA-derived lean mass was used as a surrogate measure for skeletal muscle mass. Appendicular lean mass index (ALMI) was calculated as appendicular lean mass divided by height squared (kg/m^2^); similarly, fat mass index (FMI) was calculated as whole body fat mass divided by height squared (kg/m^2^). Quantitative calcaneal ultrasound was performed on the left heel using a Lunar Achilles Insight ultrasonometer (GE Lunar, Madison, WI, USA), with measures expressed as speed of sound (SOS), broadband ultrasound attenuation (BUA) and stiffness index (SI) indicating bone quality [23]. Low trauma fractures (excluding fingers, toes, skull and face) from baseline visit until the follow-up visit for participants were ascertained using radiological reports from the radiological imaging centres servicing the region. This method of fracture ascertainment has been previously validated and described in detail [24, 25].

### Comorbidity assessment

Comorbid conditions were classified using a modified Charlson comorbidity index (CCI) [26], that took into account the number and seriousness of comorbid diseases. Using a combination of self-reported and measured data, participants were assigned weights for the following conditions: myocardial infarction, congestive heart failure, peripheral vascular disease, cerebrovascular disease, dementia, chronic pulmonary disease, connective tissue disease, ulcer disease, moderate or severe liver disease, mild liver disease, diabetes hemiplegia, moderate or severe renal disease, diabetes with end organ damage, any tumour, leukaemia, lymphoma, metastatic solid tumour, and acquired immunodeficiency syndrome (AIDS) [26].

### Lifestyle factors and socioeconomic status

Socioeconomic Indexes For Areas (SEIFA) values using 2011 Australian Bureau of Statistics (ABS) census data were determined using ABS software (these rank areas in Australia according to relative socio-economic advantage and disadvantage), which were ranked in quintiles [16] and, due to small numbers, collapsed into three groups of low (quintile 1 and 2), medium (quintile 3) and high (quintiles 4 and 5). Data regarding current smoking status (yes/no) were derived from a questionnaire. Alcohol consumption exceeding 30 g/day was derived from the Victorian Cancer Council Food Frequency Questionnaire [18].

### Statistical analyses

Descriptive characteristics of participants were presented as median (IQR) or mean (±SD) or n (%). Participants’ characteristics for frail, pre-frail, and robust groups were compared using analysis of variance (ANOVA) for parametric data, Kruskal-Wallis test for non-parametric data and chi-square test for categorical factors.

Exploratory analyses that investigated the contribution of muscle and bone parameters to frailty included binary logistic regression models where dichotomised frailty (yes/no; frailty vs pre-frail and robust combined) was considered the outcome. The area under the receiver characteristic curves (AUROCs) was also calculated. In addition, ordinal logistic regression models were used, where frailty was considered in three groups (frail, pre-frail and robust). For these statistical models, BMD, BUA, SOS, SI, ALMI and HGS measures were converted to standard deviation (SD) units using published reference data [19, 27–30]. The following characteristics were included in multivariable models: age, weight and height or BMI, smoking status, alcohol, prior fracture, FMI, SES and CCI and retained if *p* < 0.05. Somers’ D nonparametric ordinal association was calculated as a post-hoc measure in ordinal logistic models. These confounders were included as studies have shown association between these factors and frailty [6, 11, 31–33]. The estimated Somers’ D associations were adjusted for the characteristics included in the model [34]. Values closer to ±1 for Somers’ D suggest the higher contribution of a musculoskeletal factor in relation to frailty status and values tending towards zero in either direction indicate lower contribution of a musculoskeletal factor. Non-linearity assumption of the logistics models was assessed for key continuous covariates through comparing the linear trend logistic model with alternative nonlinear model assuming square and cubic nonlinear structure [35]. Boxplots were also investigated to explore the association between the frailty items and musculoskeletal parameters. Data for men and women were analysed separately. Minitab (Version 18, State College, PA, USA) and STATA (Version 16, College Station, Texas, USA) statistical software were used for statistical analyses.

## Results

### Men

The participants’ descriptive characteristics for the whole group and according to frailty status are displayed in Table 1. Frail men were older, had a higher mean BMI and were more likely to have higher mean FMI. Frail men were also under-represented in the highest SES tertile. No other differences were observed.

**Table 1:** Descriptive characteristics of participants. Data presented as median (IQR) or mean (±SD) or n (%)

	All	Frail	Pre-frail	Robust	***P***-value
**Men**	***N*** **= 347**	***N*** **= 54**	***N*** **= 188**	***N*** **= 105**	
Age (yr)	70.8 (66.1–78.6)	75.9 (68.2–82.4)	70.9 (66.2–79.2)	70.1 (64.9–74.7)	0.003
Weight (kg)	85.0 ± 13.9	88.4 ± 15.7	84.5 ± 14.2	84.2 ± 12.0	0.141
Height (cm)	173.6 ± 7.0	172.2 ± 7.2	173.6 ± 7.1	174.5 ± 6.6	0.139
BMI (kg/m^2^)	28.2 ± 4.0	29.8 ± 4.5	28.0 ± 4.0	27.6 ± 3.6	0.004
FMI (kg/m^2^)	8.6 ± 3.1	10.0 ± 3.3	8.5 ± 3.1	8.0 ± 2.8	0.001
Prior fracture	50 (14.4)	12 (22.2)	26 (13.8)	12 (11.4)	0.175
CCI	0 (0–2)	0.5 (0.0–2.0)	0 (0–2.0)	0 (0–2.0)	0.898
Femoral neck BMD (g/cm^2^)	1.0 ± 0.1	1.0 ± 0.2	0.9 ± 0.1	1.0 ± 0.1	0.421
BUA^a^ (dB/MHz)	115.4 ± 15.2	112.7 ± 15.7	115.2 ± 15.3	117.1 ± 14.8	0.219
SOS^a^ (m/s)	1564.6 ± 56.1	1556.6 ± 36.5	1564.0 ± 41.7	1569.7 ± 81.5	0.376
SI^a^	95.3 ± 19.0	90.7 ± 19.0	94.7 ± 19.1	98.8 ± 18.4	0.029
ALMI (kg/m^2^)	8.4 ± 1.0	8.0 ± 1.0	8.4 ± 1.0	8.5 ± 0.9	0.002
HGS^a^ (kg)	22.1 ± 8.5	14.4 ± 5.0	22.4 ± 9.1	25.7 ± 5.8	< 0.001
Smoking	23 (6.6)	3 (5.5)	12 (6.4)	8 (7.6)	0.867
Alcohol	49 (14.1)	7 (13.0)	23 (12.2)	19 (18.1)	0.372
SES					0.033
Low	113 (32.6)	22 (40.7)	68 (36.2)	23 (21.9)	
Medium	74 (21.3)	14 (25.9)	35 (18.6)	25 (23.8)	
High	160 (46.1)	18 (33.3)	85 (45.2)	57 (54.3)	
**Women**	***N*** **= 360**	***N*** **= 62**	***N*** **= 199**	***N*** **= 99**	
Age (yr)	71.0 (65.2–77.5)	77.5 (70.0–84.8)	70.9 (65.2–76.6)	67.5 (63.8–72.8)	< 0.001
Weight (kg)	73.8 ± 15.6	73.9 ± 18.5	74.7 ± 15.2	72.0 ± 14.6	0.378
Height (cm)	159.5 ± 6.2	156.8 ± 5.9	159.7 ± 6.0	160.9 ± 6.3	< 0.001
BMI (kg/m^2^)	29.0 ± 6.0	29.9 ± 6.9	29.3 ± 5.9	27.8 ± 5.2	0.049
FMI (kg/m^2^)	12.3 ± 4.8	12.2 ± 5.1	12.7 ± 4.8	11.5 ± 4.4	0.168
Prior fracture	95 (26.4)	24 (38.7)	50 (25.1)	21 (21.2)	0.041
CCI	0 (0–1)	1.0 (0–2.0)	0 (0–1.0)	0 (0–1.0)	< 0.001
Femoral neck BMD (g/cm^2^)	0.8 ± 0.1	0.8 ± 0.1	0.9 ± 0.1	0.8 ± 0.1	0.087
BUA^b^ (dB/MHz)	102.0 (92.9–111.5)	99.1 (92.7–108.7)	103.4 (93.6–112.2)	101.8 (91.4–111.1)	0.375
SOS^a^ (m/s)	1535.8 ± 43.5	1525.9 ± 46.1	1538.2 ± 49.9	1536.8 ± 25.6	0.233
SI^b^	77.3 (68.9–88.5)	73.5 (66.3–83.5)	79.5 (69.0–90.5)	76.5 (69.0–88.3)	0.101
ALMI^b^ (kg/m^2^)	6.6 ± 0.9	6.4 ± 0.9	6.6 ± 0.9	6.7 ± 0.7	0.194
HGS^b^ (kg)	20.6 ± 5.9	14.8 ± 5.6	20.5 ± 5.3	24.9 ± 3.5	< 0.001
Smoking	21 (5.8)	3 (4.8)	16 (8.0)	2 (2.0)	0.106
Alcohol	11 (14.1)	0 (0)	7 (3.5)	4 (4.0)	0.298
SES					0.364
Low	111 (30.8)	21 (33.9)	66 (33.2)	24 (24.2)	
Medium	142 (39.4)	27 (43.5)	74 (37.2)	41 (41.4)	
High	107 (29.7)	14 (22.6)	59 (29.6)	34 (34.3)	

*BMD* bone mineral density, *BMI* body mass index, *FMI* fat mass index, *CCI* Charlson comorbidity score, *SES* socioeconomic status, *BUA* broad- band ultrasound attenuation, *SOS* speed of sound, *SI* stiffness index, *ALMI* appendicular lean mass index, *HGS* handgrip strength

^a^Missing data for Men: - BUA, SOS and SI *n* = 2, BMD *n* = 7, HGS n = 1

^b^Missing data for Women: - BUA, SOS and SI *n* = 75, BMD *n* = 48, ALMI n = 36, HGS *n* = 16

**Fig. 2:**
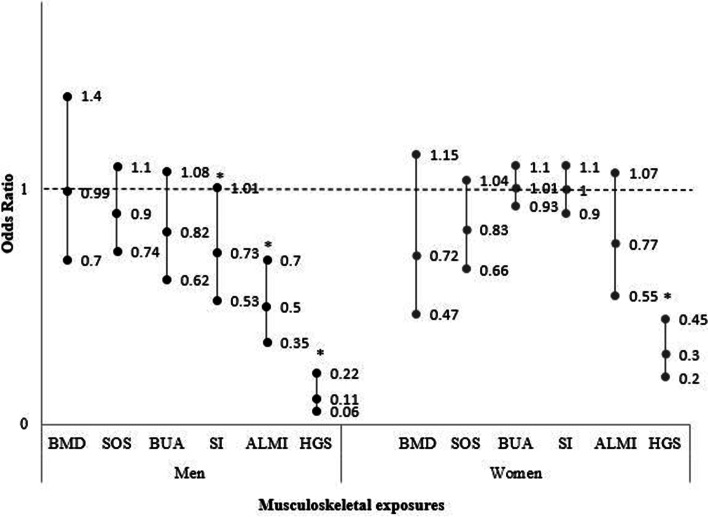
Forest plot of odds ratios (OR 95% confidence interval) of binary logistic multivariable models (which included age, weight and height or body mass index, smoking status, alcohol, prior fracture, fat mass index, socioeconomic status and Charlson comorbidity index) the contribution of musculoskeletal factors (bone and muscle) to frailty. *Footnotes: BMD = bone mineral density at femoral neck, SOS = speed of sound, BUA = broad-band ultrasound attenuation, SI = stiffness index, ALMI = appendicular lean mass index, HGS = handgrip strength. *Indicates significant difference p < 0.05

In unadjusted binary logistic models, SI, ALMI and HGS were associated with frailty in men (Table 2). The association was sustained after adjustments for relevant characteristics, where men with higher measures were less likely to be frail; SI (OR 0.73 95%CI 0.53–1.01, *p* = 0.06), ALMI (OR 0.48 95%CI 0.34–0.68, *p* < 0.001) and HGS (OR 0.11 95%CI 0.06–0.22, *p* < 0.001) (Fig. 2). No other associations were observed. Unadjusted ordinal logistic models showed similar results and these associations were sustained in adjusted models (Table 4).

**Table 2:** Binary logistic regression models for the contribution of musculoskeletal factors to frailty for men. Data presented as odds ratio (OR, 95% confidence interval (CI))

Exposures	OR (95% CI)	AUROC	*P*-value
Femoral neck BMD			
Unadjusted	0.99 (0.70–1.40)	0.52	0.96
Ultrasound SOS			
Unadjusted	0.90 (0.74–1.10)	0.58	0.29
Ultrasound BUA			
Unadjusted	0.82 (0.62–1.08)	0.55	0.16
Ultrasound SI			
Unadjusted	0.73 (0.54–1.00)	0.57	0.05
Adjusted- age, BMI	0.73 (0.53–1.01)	0.71	0.06
Final Model	*	*	*
ALMI			
Unadjusted	0.60 (0.44–0.81)	0.65	< 0.01
Adjusted- age, fat mass index	0.50 (0.36–0.70)	0.75	< 0.01
Final Model 1- age, fat mass index, SES	0.50 (0.35–0.70)	0.77	< 0.01
Final model 2-age, fracture, SES, fat mass index	0.48 (0.34–0.68)	0.77	< 0.01
HGS			
Unadjusted	0.12 (0.07–0.23)	0.83	< 0.01
Adjusted- age, height	0.12 (0.06–0.23)	0.84	< 0.01
Final Model- age, height, SES	0.11 (0.06–0.22)	0.85	< 0.01

*Final model same as adjusted model

*AUROC* area under the receiver operational characteristic curve, *BMD* bone mineral density, *BUA* broad-band ultrasound attenuation, *SOS* speed of sound, *SI* stiffness index, *ALMI* appendicular lean mass index, *HGS* handgrip strength

Muscle parameters contributed more to frailty than bone parameters (Tables 2 and 4). HGS made a greater contribution to frailty than ALMI (AUROC 0.85 and 0.77 respectively, *p* < 0.01) (Table 2). This was also observed in the ordinal regression model, where HGS had a stronger effect (Somers’ D correlation of − 0.49 (− 0.57- (−) 0.41), p < 0.01) (Table 4), meaning that HGS improved our prediction of frailty status by 49%, greater than the 36% observed for ALMI. Of the bone parameters, only SI contributed to the model and the contribution after adjusting for age and BMI was 0.71 (AUROC) (*p* = 0.06).

For femoral neck BMD, lower mean scores were associated with the following frailty items; low physical activity, fatigue, slowness and low HGS. Weight loss was associated with higher mean femoral neck BMD. Similar results were observed for ALMI and heel ultrasound measures (BUA, SOS, SI), where lower mean scores were associated with low physical activity, fatigue, slowness and low HGS, while the inverse was true for weight loss. (Supplementary Fig. 1). Overall, for low physical activity, SI median scores had the greatest percentage decrease (13.0%) in median score between the yes and no groups compared to the other musculoskeletal parameters. For fatigue, ALMI had the greatest percentage decrease (1.7%) while for slowness, SI had the greatest percentage decrease (10.1%). For low HGS, ALMI had the greatest percentage decrease (5.7%). Lastly, for weight loss, SI had the greatest percentage increase of 7.4% between the no and yes groups. In correlation tests for frailty items, slowness (as measured by TUG) showed a strong correlation (Supplementary Table 1) and thus, TUG was modelled as binary logistic regression models. The TUG models were inferior in the AUROC for the ALMI model (from 0.77 to 0.76 (*p* < 0.001)) and the HGS model (from 0.85 to 0.79 (p < 0.001)).

### Women

Frail women were also older and shorter and had a higher mean BMI than pre-frail and robust women. They were more likely to have a prior fracture and a higher number of comorbid conditions (Table 1).

In unadjusted binary logistic models, BMD and HGS were associated with frailty (Table 3). After adjustments for age, weight and height, only HGS was associated with frailty, where women with greater HGS were less likely to be frail (OR 0.30, (95%CI 0.20–0.45) *p* < 0.001) (Fig. 2). No other associations were observed (Table 3). In unadjusted ordinal logistic models, HGS was associated with frailty, while an association that did not achieve statistical significance was observed with ALMI. After adjustments for age, height, FMI and SES, HGS and ALMI were associated with frailty (Table 4).

**Table 3:** Binary logistic regression models for the contribution of musculoskeletal factors to frailty for women. Data presented as odds ratio (OR, 95% confidence interval (CI))

Exposures	OR (95% CI)	AUROC	*P*-value
Femoral neck BMD			
Unadjusted	0.68 (0.48–0.97)	0.60	0.04
Adjusted- age, weight, height	0.72 (0.47–1.15)	0.75	0.15
Final Model	*	*	*
Ultrasound SOS			
Unadjusted	0.83 (0.66–1.04)	0.60	0.10
Ultrasound BUA			
Unadjusted	1.01 (0.93–1.10)	0.46	0.85
Ultrasound SI			
Unadjusted	1.00 (0.90–1.10)	0.58	0.99
ALMI			
Unadjusted	0.77 (0.55–1.07)	0.57	0.12
HGS			
Unadjusted	0.23 (0.16–0.35)	0.81	< 0.01
Adjusted- age, height	0.30 (0.20–0.45)	0.83	< 0.01
Final Model	*	*	*

*Final model same as adjusted model

*AUROC* Area under the receiver operational characteristic curve, *BMD* bone mineral density, *BUA* broad-band ultrasound attenuation, *SOS* speed of sound, *SI* stiffness index, *ALMI* appendicular lean mass index, *HGS* handgrip strength

**Table 4:** Ordinal logistic regression models for the contribution of musculoskeletal factors to frailty (categorised as frail, pre-frail and robust) for men and women. Data presented as OR (95%CI) and Somers D correlation (95%CI)

	Men			Women		
Exposures	Ordinal OR (95% CI)	Somers D correlation (95%CI)	***P***-value	Ordinal OR (95% CI)	Somers D correlation (95%CI)	***P***-value
**Femoral neck BMD**						
Unadjusted	0.44 (0.09–2.15)		0.31	0.41 (0.07–2.28)		0.31
**SOS**						
Unadjusted	1.00 (0.99–1.00)		0.14	1.00 (0.99–1.00)		0.32
**BUA**						
Unadjusted	0.99 (0.98–1.00)		0.09	1.00 (1.00–1.01)		0.29
**SI**						
Unadjusted	0.99 (0.97–1.00)		0.01	1.00 (1.00–1.01)		0.31
Adjusted- age, BMI	0.99 (0.98–1.00)		0.01			
Final Model- age, BMI,SES	0.99 (0.98–1.00)	−0.33 (−0.43- (−)0.22)	0.02			
**ALMI**						
Unadjusted	0.72 (0.58–0.88)		0.00	0.81 (0.64–1.03)		0.09
Adjusted- age, fat mass index	0.67 (0.54–0.84)		0.00	0.58 (0.41–0.82)		0.00
Final Model-age, fat mass index, SES	0.68 (0.54–0.86)	−0.36 (− 0.45– (−)0.26)	0.00	0.56 (0.40–0.80)	−0.41 (− 0.51– (−)0.31)	0.00
**HGS**						
Unadjusted	0.35 (0.26–0.47)		0.00	0.24 (0.18–0.32)		0.00
Adjusted- age, height	0.36 (0.26–0.49)		0.00	0.29 (0.21–0.39)		0.00
Final Model- age, height, SES	0.33 (0.24–0.46)	−0.49 (−0.57- (−)0.41)	0.00	0.28 (0.21–0.39)	−0.62 (− 0.69- (−)0.54)	0.00

*BMD* bone mineral density, *BUA* broad-band ultrasound attenuation, *SOS* speed of sound, *SI* stiffness index, *ALMI* appendicular lean mass index, *HGS* handgrip strength

*Robust was the baseline strata and the odds ratio (OR) correspond to one category increase in frailty status

In binary logistic regression models, only HGS was observed to contribute to the model (AUROC 0.83, p < 0.001) (Table 3). However, when frailty was considered as three groups, ALMI was also observed to contribute to the model (p < 0.001). In these ordinal regression models, HGS had a stronger contribution to frailty than did ALMI (Table 4).

Overall boxplots that explored the association between the frailty items and musculoskeletal parameters did not reveal any apparent associations or trends across the musculoskeletal parameters. (Supplementary Fig. 2).

In correlation tests for frailty items, slowness (as measured by TUG) showed a strong correlation (Supplementary Table 2) and thus, TUG was modelled as binary logistic regression models. The TUG models were inferior compared with the HGS models (AUROC decrease from 0.83 to 0.74 (*p* = 0.001)).

For all models with significant exposure (i.e., for men: SI, ALMI and HGS and for women: ALMI and HGS), an inclusive model that included important potential confounders irrespective the *p*-values were employed. A link-test was utilised to test for any potential regression model specification error [36]. The link-test tests the idea that if a regression equation is properly specified no additional independent variables that are significant can be found except by chance. In addition, the square and cubic terms were added to the logistics models to test for potential nonlinearity. In conclusion none of the link-tests or testing the joint square and cubic terms were significant, and as such we did not detect any strong evidence of lack of regression goodness of fit. Furthermore, multivariate regression models that included necessary covariates were constructed, however, the conclusion did not substantially change by these adding additional factors (Supplementary Table 2-14).

## Discussion

Our study suggests that muscle parameters make a greater contribution to frailty compared to bone parameters for men and women. For men, when frailty was considered as a binary outcome, muscle measures (lean mass and HGS) contributed to models of frailty more than bone measures (BMD, BUA, SOS and SI). Similar results were observed when frailty was considered in the three groups (robust, pre-frail and frail). For women, considering frailty as a binary outcome, only HGS contributed to the models. However, when frailty was considered in three groups, lean mass and HGS contributed to the models. No bone measures were observed to contribute to frailty in women.

The clinical signs of frailty include weight loss, reduced physical activity, balance and gait speed, reduced cognitive function and altered state of nutrition, and our data suggests contributions by parameters of sarcopenia and a lesser contribution from osteoporosis [8]. In a study of 3231 European men aged 40-79 yr, pre-frail and frail men had lower SOS and BUA and frail men had a lower femoral BMD compared to robust men [37]. Our study indicates that SI, a mathematical combination of SOS and BUA, was associated with frailty in men [23]. No association was observed with femoral neck BMD in our study which concurs with another study including 392 community dwelling men age 58–95 yr which reported that although femoral neck BMD was associated with frailty, this association was attenuated after adjustment for age [38]. Another study of 235 community-dwelling older women observed frailty, defined by the Fried phenotype and a self-reported Vulnerable Elders Survey (VES-13), was not correlated with hip or spine BMD, although frail women (defined by VES-13) had lower hip and spine BMD after one year [14]. While in a longitudinal study of 75 yr old community-dwelling women, frailty was associated with low BMD and higher mortality risk [39]. Similar observations were made in another study including 257 community-dwelling participants of the Women Health and Aging Study II, with higher rates of frailty among those with severe osteoporosis/osteopenia as defined in terms of low BMD [13].

Sarcopenia, defined as age-related loss of muscle mass and a decline in muscle function [40] has overlapping constructs with frailty [41–43]. Overall, our data were observed to suggest that muscle parameters in both men and women as major contributors to frailty as compared to bone parameters. Indeed HGS contributed the most to the models compared to other musculoskeletal parameters, which is likely to reflect the consideration of HGS as a marker of weakness in the Fried definition of frailty [6]. The use of a different tool such as the Rockwood frailty index of deficit accumulation [7] may present different results as the focus of this tool is both physical aspects of frailty as well as psychosocial domains. At present, sufficient data were not available in our study to calculate the frailty index of deficit accumulation to test this theory.

During ageing there is concomitant deterioration of muscle and bone that may manifest in the form of frailty [12, 44]. Although both sarcopenia and osteoporosis contribute to frailty secondary to age-related changes in body composition, hormonal imbalance and chronic inflammation [8, 12, 45], parameters of osteoporosis appeared to contribute less than measures of sarcopenia. Our models, at least in men, suggest that individuals with marked deficits in both bone and muscle (osteosarcopenia) are more likely to be frail. However, with advancing age, there is an integrated and progressive decline of both muscle and bone [9], making it difficult to make a clear distinction of their individual contributions to frailty.

The strengths of this study include the use of objective measures for bone and muscle parameters and that participants were from an unselected population. Our study also had some limitations including the use of a modified Fried phenotype, some self-reported data and the use of DXA-derived lean mass as a surrogate for muscle mass. Although different dynamometers were used in assessing HGS in men and women, the data were harmonised by converting Vernier values into Jamar equivalents. Participants were pre-dominantly Caucasian, thus, our results may not be applicable to other populations. As data were cross-sectional, no inferences can be drawn about causality.

## Conclusion

From the parameters measured in this study, our data suggest that muscle deficits may contribute more to frailty than bone deficits (however due to the cross-sectional nature of the study, causality cannot be conferred), and this was observed for both men and women. From these observations, interventions for addressing the trajectory of frailty might be targeted to improve muscle health, although this conjecture would need to be investigated using prospective data from a longitudinal study as well as data from intervention studies targeting muscle and/or bone. We suggest further studies should be performed using other definitions frailty and in larger diverse populations to obtain a more comprehensive overview of the contributions of musculoskeletal parameters to frailty.

## Supplementary Information

**Additional file 1: Supplementary Fig. 1**. Association between frailty items in the Fried frailty phenotype and musculoskeletal parameters in men. Frailty items; 1 = Low physical activity, 2 = fatigue, 3 = weight loss, 4 = slowness, 5 = low handgrip strength, Heel Ultrasound measures; BUA = broadband ultrasound attenuation, SOS = speed of sound, SI = stiffness index. **Supplementary Fig. 2**. Association between frailty items in the Fried frailty phenotype and musculoskeletal parameters in women. Frailty items; 1 = Low physical activity, 2 = fatigue, 3 = weight loss, 4 = slowness, 5 = low handgrip strength, Heel Ultrasound measures; BUA = broadband ultrasound attenuation, SOS = speed of sound, SI = stiffness index. **Supplementary Table 1**. Spearman correlation of frailty items with frailty in men. **Supplementary Table 2**. Spearman correlation of frailty items with frailty in women. Table 3. Model 1 Femoral BMD. Table 4. Model 2 BUA. Table 5 Model 3 SI. Table 6. Model 4 SOS. Table 7. Model 5 ALMI. Table 8. Model 6 HGS. Table 9. Model 1 Femoral BMD. Table 10 Model 2 BUA. Table 11 Model 3 SI. Table 12 Model 4 SOS. Table 13 Model 5 ALMI. Table 14. Model 6 HGS.

## Data Availability

The datasets generated and/or analysed during the current study are not publicly available due to data privacy rules but are available from the corresponding author on reasonable request.

## Abbreviations

AIDS: Immunodeficiency syndrome
ABS: Australian Bureau of Statistics
ALMI: Appendicular lean mass index
ANOVA: Analysis of variance
AUROCs: Area under the receiver characteristic curves
BMD: Lower bone mineral density
BMI: Body mass index
BUA: Broadband ultrasound attenuation
CCI: Charlson Comorbidity Index
DXA: Dual-energy X-ray absorptiometry
FMI: Fat mass index
GOS: Geelong Osteoporosis Study
HGS: Handgrip Strength
IQR: Interquartile range
OR: Odds Ratio
SD: Standard deviation
SEIFA: Socioeconomic Indexes For Areas
SES: Social economic status
SI: Stiffness index
SOS: Speed of Sound
TUG: Timed Up and Go
VES-13: Vulnerable Elders Survey
