# Supplementary material for: The contribution of musculoskeletal factors to physical frailty: a cross-sectional study
Source: BMC Musculoskelet Disord. 2021 Nov 1;22:921. doi: 10.1186/s12891-021-04795-4 (PMC8561908; doi:10.1186/s12891-021-04795-4)
Supplement: Supplementary file 1 — Additional file 1: Supplementary Fig. 1. Association between frailty items in the Fried frailty phenotype and musculoskeletal parameters in men. Frailty items; 1 = Low physical activity, 2 = fatigue, 3 = weight loss, 4 = slowness, 5 = low handgrip strength, Heel Ultrasound measures; BUA = broadband ultrasound attenuation, SOS = speed of sound, SI = stiffness index. Supplementary Fig. 2. Association between frailty items in the Fried frailty phenotype and musculoskeletal parameters in women. Frailty items; 1 = Low physical activity, 2 = fatigue, 3 = weight loss, 4 = slowness, 5 = low handgrip strength, Heel Ultrasound measures; BUA = broadband ultrasound attenuation, SOS = speed of sound, SI = stiffness index. Supplementary Table 1. Spearman correlation of frailty items with frailty in men. Supplementary Table 2. Spearman correlation of frailty items with frailty in women. Table 3. Model 1 Femoral BMD. Table 4. Model 2 BUA. Table 5 Model 3 SI. Table 6. Model 4 SOS. Table 7. Model 5 ALMI. Table 8. Model 6 HGS. Table 9. Model 1 Femoral BMD. Table 10 Model 2 BUA. Table 11 Model 3 SI. Table 12 Model 4 SOS. Table 13 Model 5 ALMI. Table 14. Model 6 HGS. [file 12891_2021_4795_MOESM1_ESM.pdf]

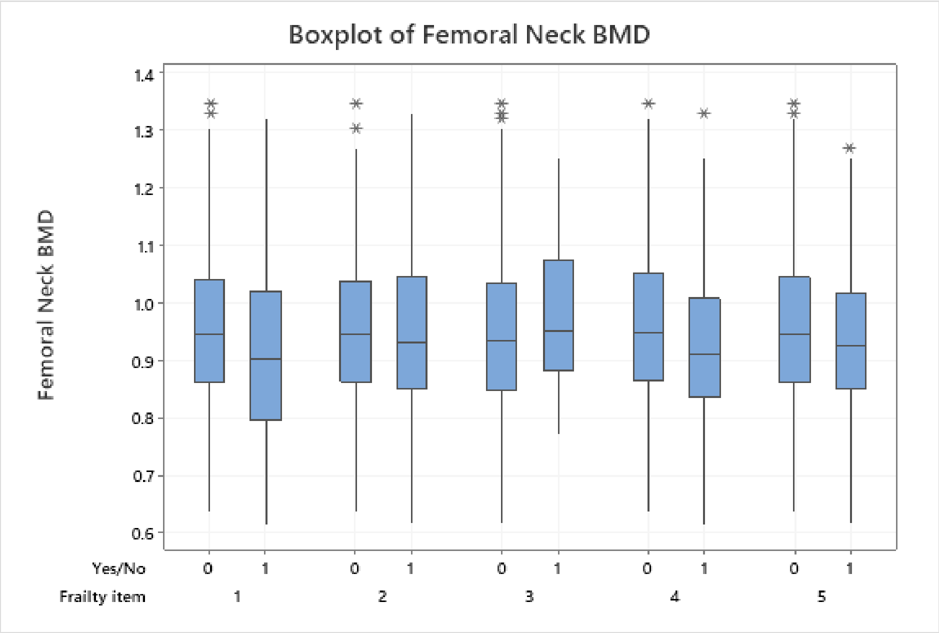
Boxplot of Femoral Neck BMD
Femoral Neck BMD
1.4
1.3
1.2
1.1
1.0
0.9
0.8
0.7
0.6
Yes/No 0 1 0 1 0 1 0 1 0 1
Frailty item 1 2 3 4 5

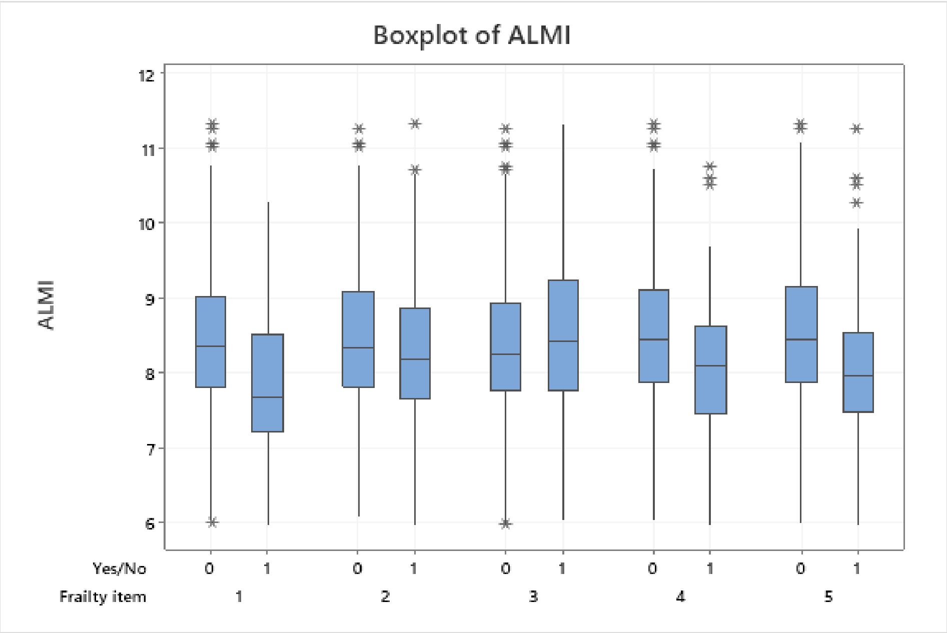
Boxplot of ALMI
ALMI
12
11
10
9
8
7
6
Yes/No 0 1 0 1 0 1 0 1 0 1
Frailty item 1 2 3 4 5

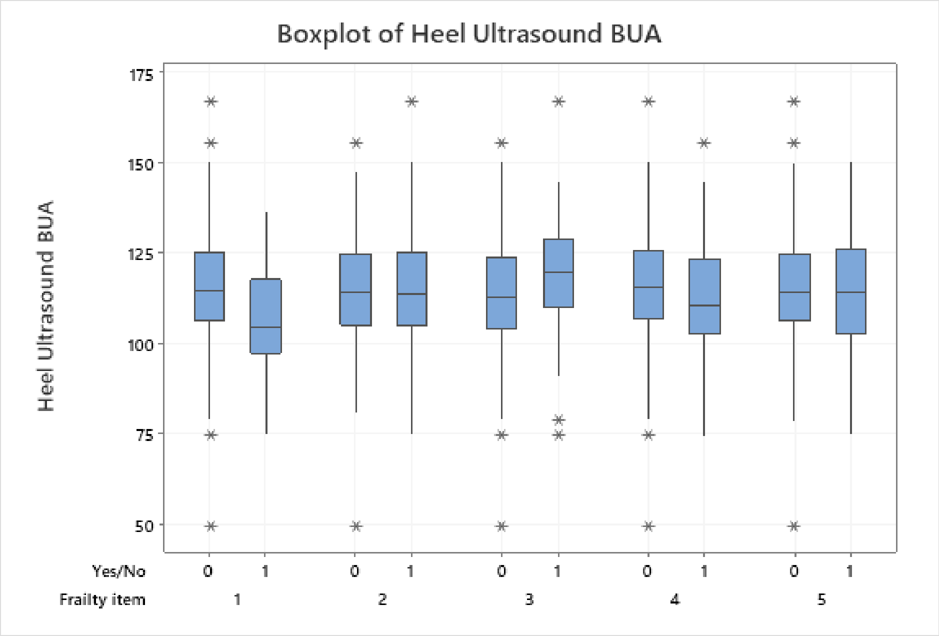
Boxplot of Heel Ultrasound BUA
Heel Ultrasound BUA
175
150
125
100
75
50
Yes/No 0 1 0 1 0 1 0 1 0 1
Frailty item 1 2 3 4 5

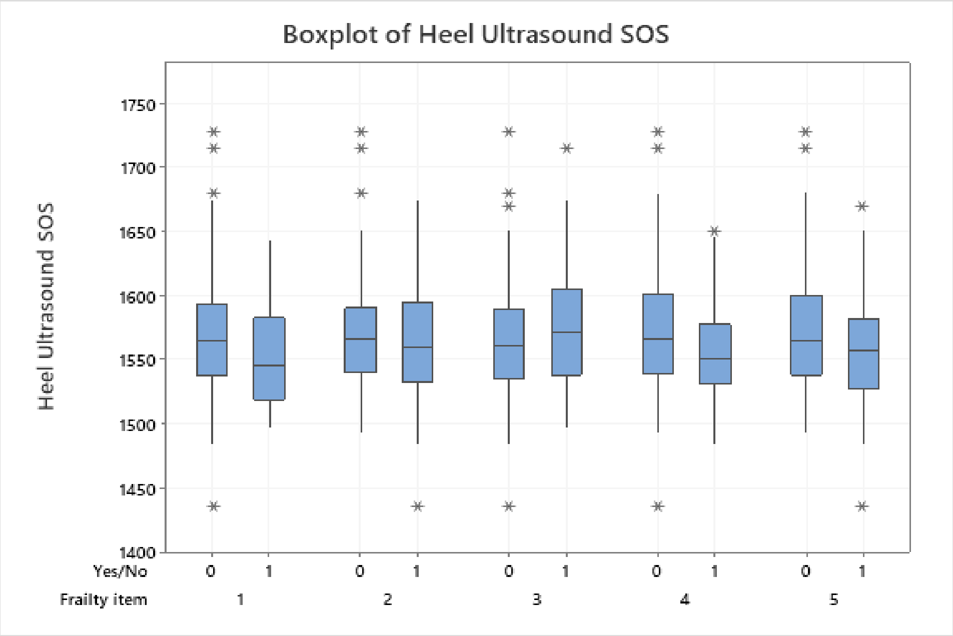
Boxplot of Heel Ultrasound SOS
Heel Ultrasound SOS
1750
1700
1650
1600
1550
1500
1450
1400
Yes/No 0 1 0 1 0 1 0 1 0 1
Frailty item 1 2 3 4 5

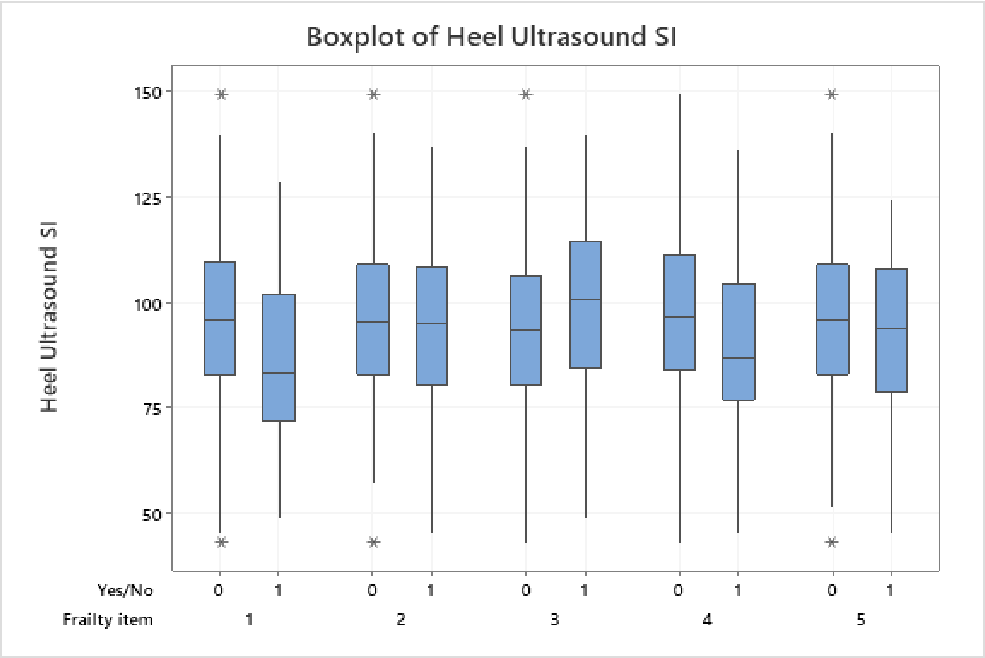

Supplementary Figure 1 Association between frailty items in the Fried frailty phenotype and musculoskeletal parameters in men.

Frailty items; 1= Low physical activity, 2=fatigue, 3= weight loss, 4= slowness, 5= low handgrip strength

Heel Ultrasound measures; BUA= broadband ultrasound attenuation, SOS= speed of sound, SI= stiffness index

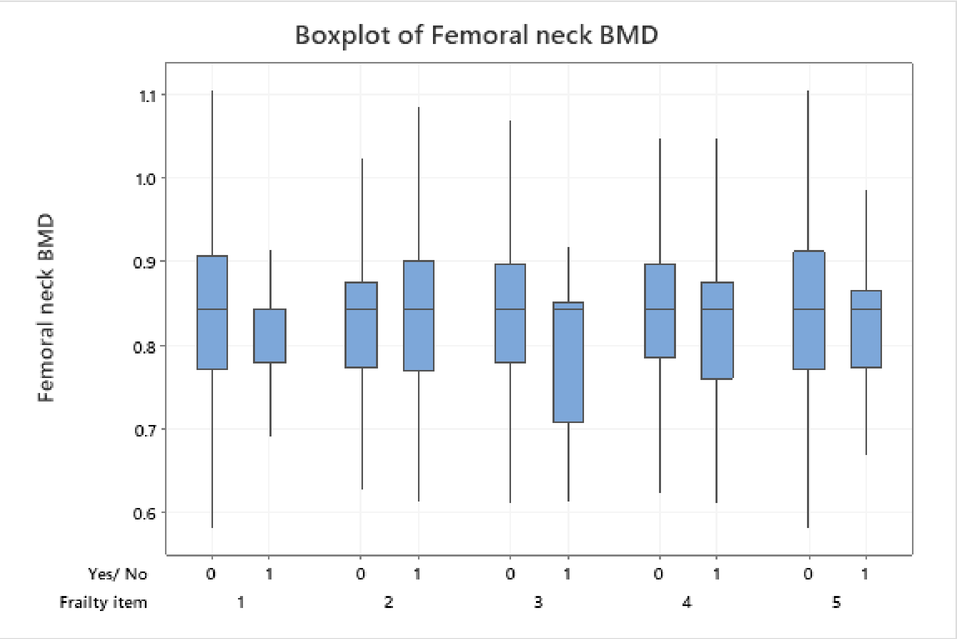
Boxplot of Femoral neck BMD
Femoral neck BMD
1.1
1.0
0.9
0.8
0.7
0.6
Yes/ No 0 1 0 1 0 1 0 1 0 1
Frailty item 1 2 3 4 5

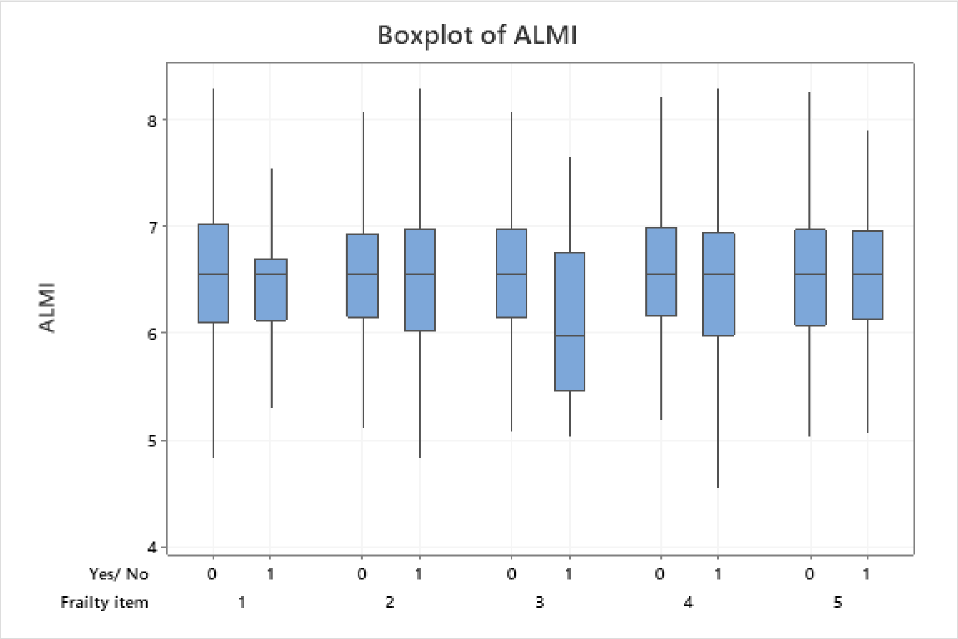
Boxplot of ALMI
ALMI
8
7
6
5
4
Yes/ No 0 1 0 1 0 1 0 1 0 1
Frailty item 1 2 3 4 5

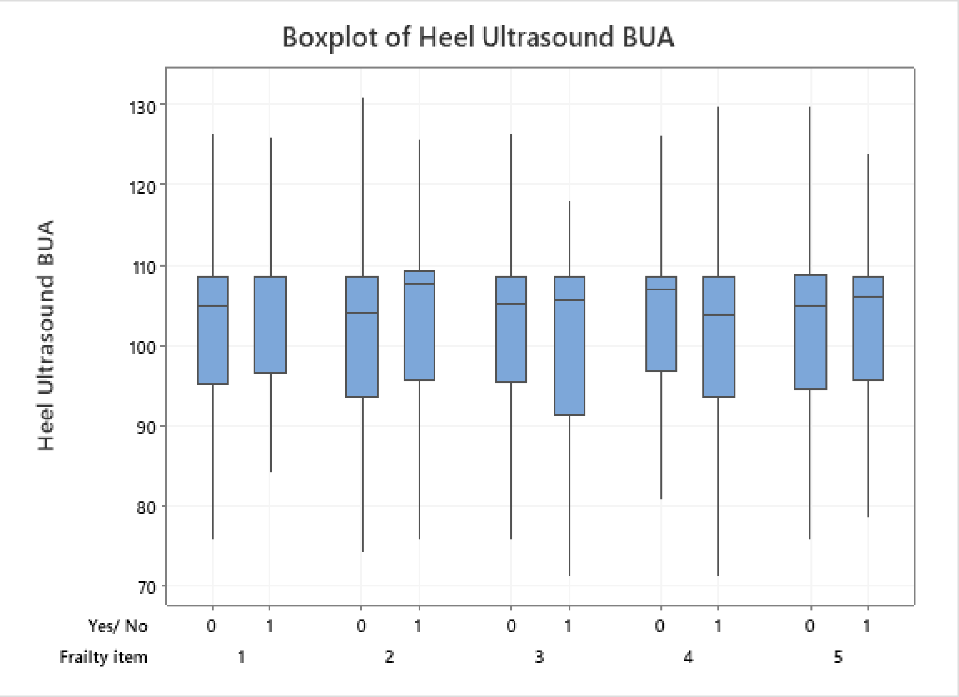
Boxplot of Heel Ultrasound BUA
Heel Ultrasound BUA
130
120
110
100
90
80
70
Yes/ No 0 1 0 1 0 1 0 1 0 1
Frailty item 1 2 3 4 5

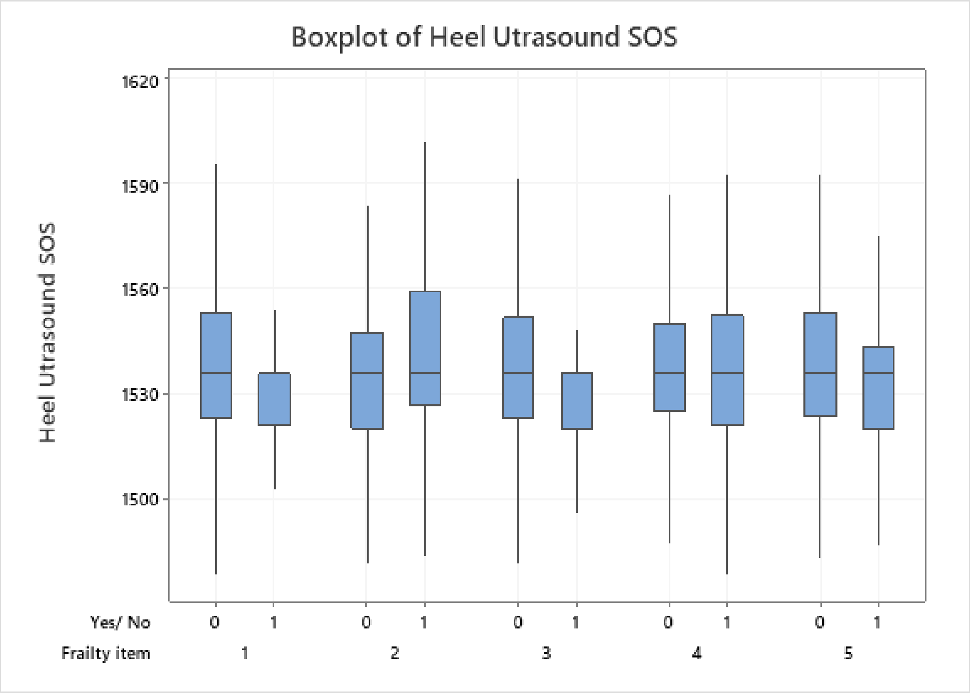
Boxplot of Heel Utrasound SOS
Heel Utrasound SOS
1620
1590
1560
1530
1500
Yes/ No 0 1 0 1 0 1 0 1 0 1
Frailty item 1 2 3 4 5

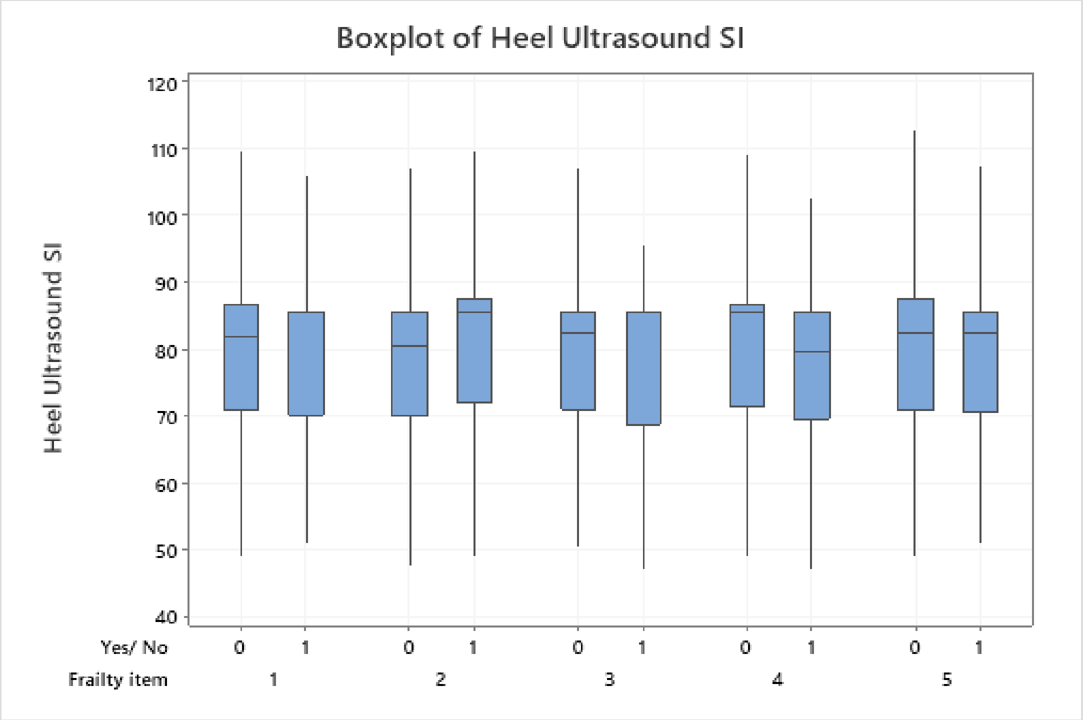

Supplementary Figure 2 Association between frailty items in the Fried frailty phenotype and musculoskeletal parameters in women.

Frailty items; 1= Low physical activity, 2=fatigue, 3= weight loss, 4= slowness, 5= low handgrip strength

Heel Ultrasound measures; BUA= broadband ultrasound attenuation, SOS= speed of sound, SI= stiffness index

Supplementary Table 1 Spearman correlation of frailty items with frailty in men

	Frailty	Low physical activity	Weight loss	Fatigue	Slowness
Low physical activity	0.460				
Weight loss	0.384	0.085			
Fatigue	0.598	0.176	0.273		
Slowness	0.570	0.330	-0.004	0.083	
Low HGS*	0.547	0.250	0.051	-0.015	0.361

*HGS= Handgrip strength

Supplementary Table 2 Spearman correlation of frailty items with frailty in women

	Frailty	Weight loss	Fatigue	Low physical activity	Slowness
Weight Loss	0.316				
Fatigue	0.554	0.204			
Low physical activity	0.572	0.212	0.101		
Slowness	0.652	0.103	0.106	0.295	
HGS	0.617	0.078	0.020	0.257	0.261

Men Logistic regression models including all covariates

Table 3 Model 1 Femoral BMD

Covariate	Odds Ratio	P-value	95% Confidence Interval
Femoral Neck BMD	0.92	0.68	0.63-1.34
Age	1.05	0.009	1.01-1.09
BMI	1.12	0.004	1.04-1.22
Fracture	2.03	0.08	0.93-4.46
SES	0.90	0.80	0.39-2.04
Alcohol	1.18	0.74	0.46-3.03
Smoking	1.02	0.98	0.27-3.90
CCI	1.25	0.79	0.26-6.07

Table 4 Model 2 BUA

Covariate	Odds Ratio	P-value	95% Confidence Interval
BUA	0.85	0.29	0.63-1.14
Age	1.05	0.01	1.01-1.09
BMI	1.12	0.002	1.04-1.21
Fracture	1.64	0.22	0.74-3.63
SES	0.83	0.64	0.37-1.85
Alcohol	1.11	0.83	0.43-2.82
Smoking	1.01	1.00	0.27-3.90
CCI	1.15	0.86	0.25-5.40

Table 5 Model 3 SI

Covariate	Odds Ratio	P-value	95% Confidence Interval
SI	0.77	0.14	0.55-1.09
Age	1.04	0.02	1.01-1.08
Weight	1.03	0.002	1.01-1.06
Height	0.94	0.01	0.89-0.99
Fracture	1.51	0.31	0.68-3.34
SES	0.88	0.77	0.39-1.99
Alcohol	1.09	0.86	0.43-2.75
Smoking	0.94	0.93	0.25-3.62
CCI	1.23	0.80	0.26-5.89

Table 6 Model 4 SOS

Covariate	Odds Ratio	P-value	95% Confidence Interval
SOS	0.93	0.48	0.76-1.14
Age	1.05	0.02	1.01-1.08
Weight	1.04	0.004	1.01-1.06
Height	0.94	0.01	0.89-0.99
Fracture	1.69	0.19	0.78-3.68
SES	0.85	0.70	0.38-1.92
Alcohol	1.11	0.82	0.44-2.81
Smoking	0.99	0.99	0.26-3.78
CCI	1.22	0.81	0.252-5.88

Table 7 Model 5 ALMI

Covariate	Odds Ratio	P-value	95% Confidence Interval
ALMI	0.46	0.000	0.32-0.66
Age	1.03	0.10	0.99-1.07
Fracture	2.40	0.04	1.06-5.42
SES	0.89	0.79	0.38-2.07
Alcohol	1.01	0.99	0.38-2.68
Smoking	0.67	0.58	0.17-2.69
CCI	2.30	0.31	0.47-11.30
FMI	1.32	0.000	1.18-1.48

Table 8 Model 6 HGS

Covariate	Odds Ratio	P-value	95% Confidence Interval
HGS	0.19	0.000	0.11-0.32
Age	1.00	0.83	0.96-1.05
Height	1.02	0.26	0.98-1.08
Fracture	1.77	0.19	0.75-4.17
SES	0.83	0.69	0.34-2.04
Alcohol	0.98	0.96	0.36-2.63
Smoking	0.76	0.73	0.17-3.38
CCI	2.90	0.22	0.54-15.65

**BMD= Bone mineral density, BMI= Body mass index, SES= Socioeconomic status, CCI= Charlson comorbidity index, BUA=Broadband ultrasound attenuation, SI= Stiffness index, SOS= Speed of sound, ALMI= Appendicular lean mass index, FMI= Fat mass index, HGS= Hand grip strength

Women Logistic regression models including all covariates

Table 9 Model 1 Femoral BMD

Covariate	Odds Ratio	P-value	95% Confidence Interval
Femoral Neck BMD	0.75	0.20	0.48-1.17
Age	1.10	0.000	1.05-1.16
BMI	1.09	0.02	1.01-1.18
Fracture	1.25	0.59	0.56-2.79
SES	0.84	0.68	0.36-1.95
Alcohol	*	*	*
Smoking	1.84	0.40	0.45-7.59
CCI	2.2	0.55	0.17-29.12

Table 10 Model 2 BUA

Covariate	Odds Ratio	P-value	95% Confidence Interval
BUA	0.92	0.59	0.68-1.24
Age	1.12	0.000	1.06-1.18
BMI	1.08	0.02	1.01-1.15
Fracture	0.93	0.87	0.37-2.31
SES	1.02	0.97	0.43-2.39
Alcohol	*	*	*
Smoking	1.95	0.35	0.48-7.91
CCI	5.30	0.02	1.30-21.56

Table 11 Model 3 SI

Covariate	Odds Ratio	P-value	95% Confidence Interval
SI	0.73	0.19	0.45-1.17
Age	1.12	0.000	1.05-1.17
BMI	1.07	0.03	1.01-1.15
Fracture	0.73	0.51	0.29-1.86
SES	0.98	0.96	0.42-2.30
Alcohol	*	*	*
Smoking	1.76	0.43	0.44-7.10
CCI	3.69	0.06	0.94-14.49

Table 12 Model 4 SOS

Covariate	Odds Ratio	P-value	95% Confidence Interval
SOS	0.81	0.15	0.60-1.08
Age	1.11	0.00	1.05-1.17
Weight	1.03	0.03	1.00-1.06
Height	0.93	0.03	0.87-0.99
Fracture	0.80	0.64	0.32-2.01
SES	1.03	0.94	0.32-2.01
Alcohol	*	*	*
Smoking	1.71	0.46	0.41-7.21
CCI	5.69	0.02	1.39-23.32

Table 13 Model 5 ALMI

Covariate	Odds Ratio	P-value	95% Confidence Interval
ALMI	0.02	0.01	0.00-0.42
Age	1.13	0.000	1.07-1.18
Fracture	1.04	0.92	0.47-2.27
SES	1.10	0.82	0.47-2.56
Alcohol	*	*	*
Smoking	2.00	0.33	0.50-7.96
CCI	3.20	0.37	0.25-40.44
FMI	1.16	0.005	1.04-1.28

Table 14 Model 6 HGS

Covariate	Odds Ratio	P-value	95% Confidence Interval
HGS	0.49	0.000	0.36-0.66
Age	1.05	0.05	1.00-1.09
Height	0.96	0.14	0.90-1.01
Fracture	1.00	1.00	0.47-2.13
SES	0.75	0.48	0.33-1.68
Alcohol	*	*	*
Smoking	0.88	0.86	0.21-3.66
CCI	4.57	0.27	0.31-68.39

*Denotes not enough data to run analysis

**BMD= Bone mineral density, BMI= Body mass index, SES= Socioeconomic status, CCI= Charlson comorbidity index, BUA=Broadband ultrasound attenuation, SI= Stiffness index, SOS= Speed of sound, ALMI= Appendicular lean mass index, FMI= Fat mass index, HGS= Hand grip strength

Men Data

Exposure: ALMI

Step 1: The inclusive logistic model is presented.

```
. logistic fraility_dich rALM Age BMI i.Fracture i.SES i.Smoking i.CharlsonComorbidity Fatmassindex, or
base

Logistic regression                                        Number of obs =    345

                                                           LR chi2(13)   = 58.97

                                                           Prob > chi2   = 0.0000

Log likelihood = -120.19782                                Pseudo R2     = 0.1970

------------------------------------------------------------------------------------
  fraility_dich | Odds ratio   Std. err.      z    P>|z|     [95% conf. interval]
----------------+-------------------------------------------------------------------
           ALMI |   .2983879   .0776547    -4.65   0.000     .1791671    .4969404
            Age |   1.030198   .0202046     1.52   0.129     .9913489    1.070569
            BMI |   1.373065   .1594045     2.73   0.006     1.093633    1.723893
                |
       Fracture |
             0  |          1  (base)
             1  |   2.394882   .9984614     2.09   0.036     1.057805    5.422038
                |
            SES |
             1  |          1  (base)
             2  |   .9545276   .4167347    -0.11   0.915     .4056623    2.246013
             3  |   .4963864    .197493    -1.76   0.078     .2275934     1.08263
                |
        Smoking |
             0  |          1  (base)
             1  |   .8226749   .5841438    -0.27   0.783     .2045639    3.308473
                |
CharlsonComor~y |
```

```
           0  |          1  (base)
           1  |    .983323   .4261215    -0.04   0.969     .4205613    2.299127
           2  |   .6630003   .3317627    -0.82   0.411     .2486428    1.767875
           3  |   1.518698   1.028593     0.62   0.537     .4026841    5.727674
           4  |   1.175665   1.108821     0.17   0.864     .1851309     7.46601
           5  |    .768004   .9979386    -0.20   0.839     .0601615    9.804114
           6  |          1  (empty)
           8  |          1  (empty)
              |
Fatmassindex  |   .9517502   .1209597    -0.39   0.697     .7418946    1.220966
       _cons  |   2.05e-06   6.06e-06    -4.43   0.000     6.25e-09    .0006725
---------------------------------------------------------------------------------
Note: _cons estimates baseline odds.
```

Step 2: The link test.

```
. linktest, nolog

Logistic regression                                     Number of obs =    345
                                                        LR chi2(2)    =  51.55
                                                        Prob > chi2   = 0.0000
Log likelihood = -123.90637                             Pseudo R2     = 0.1722

------------------------------------------------------------------------------
frailty_dich | Coefficient  Std. err.      z    P>|z|     [95% conf. interval]
-------------+----------------------------------------------------------------
       *_hat |   .6437314    .364733     1.76   0.078    -.0711321    1.358595
     ** _hatsq |  -.1312012   .1259561   -1.04   0.298    -.3780707    .1156683
       _cons |  -.1192326   .2886283    -0.41   0.680    -.6849336    .4464685
------------------------------------------------------------------------------
```

***The linear predicted value has a low p-value indicating an acceptable predictability.**

****The linear predicted value squared is not significant, indicating no evidence of misspecification.**

Step 3: Including square and cubic terms into the regression model and performing the joint square and cubic terms test.

```
. logistic frailty_dich rALM Age BMI i.Fracture i.SES  i.Alcohol i.Smoking i.CharlsonComorbidity
Fatmassindex xbhsq_rALM xbhcu_rALM, or base
. test xbhsq_rALM xbhcu_rALM
 ( 1)  [frailty_dich]xbhsq_rALM = 0
 ( 2)  [frailty_dich]xbhcu_rALM = 0

           chi2( 2) =    4.15

         Prob > chi2 =    0.1259
```

(Non-significant, no evidence of misspecification)

Exposure: SI

Step 1.

```
. logistic fraility_dich SI Age BMI i.Fracture i.SES i.Smoking i.CharlsonComorbidity Fatmassindex, or
base

Logistic regression                                     Number of obs =    343
                                                        LR chi2(13)   =  30.91
                                                        Prob > chi2   = 0.0035
Log likelihood = -133.88401                             Pseudo R2     = 0.1035

--------------------------------------------------------------------------------
  fraility_dich | Odds ratio   Std. err.      z    P>|z|     [95% conf. interval]
----------------+---------------------------------------------------------------
             SI |   .7937888   .1387124    -1.32   0.186     .5635856    1.118021
            Age |   1.044892   .0194385     2.36   0.018      1.00748    1.083694
            BMI |   .9884019   .0811789    -0.14   0.887       .84144    1.161031
                |
       Fracture |
             0  |          1  (base)
             1  |   1.703154   .7027375     1.29   0.197     .7586472    3.823558
                |
            SES |
             1  |          1  (base)
             2  |    .823384   .3388971    -0.47   0.637     .3674988    1.844799
             3  |    .477382   .1765616    -2.00   0.046     .2312309    .9855672
                |
        Smoking |
             0  |          1  (base)
             1  |   .8888654   .6160963    -0.17   0.865     .2284786    3.458012
                |
CharlsonComor~y |
             0  |          1  (base)
```

1	.7780867	.3280084	-0.60	0.552	.3405631	1.7777
2	.6287387	.2918881	-1.00	0.318	.2531076	1.561835
3	1.131058	.7230809	0.19	0.847	.3230836	3.95963
4	1.669593	1.327067	0.64	0.519	.3515906	7.928369
5	.7488349	.959847	-0.23	0.821	.0607193	9.23518
6	1	(empty)				
8	1	(empty)				
Fatmassindex	1.217612	.135668	1.77	0.077	.9787392	1.514785
_cons	.0020212	.0043929	-2.85	0.004	.0000285	.1430984

Note: _cons estimates baseline odds.

Step 2

```
. linktest, nolog

Logistic regression                                     Number of obs =    343
                                                        LR chi2(2)    =  33.62
                                                        Prob > chi2   = 0.0000
Log likelihood = -132.52831                             Pseudo R2     = 0.1126

------------------------------------------------------------------------------
frailty_dich | Coefficient  Std. err.      z    P>|z|     [95% conf. interval]
-------------+----------------------------------------------------------------
     * _hat |   .2168663    .521078     0.42   0.677    -.8044279     1.23816
  ** _hatsq |  -.2874587   .1828032    -1.57   0.116    -.6457465     .070829
      _cons |  -.3557897   .3919846    -0.91   0.364    -1.124065    .4124861
------------------------------------------------------------------------------
```

The linear predicted value has a ? p-value indicating an acceptable predictability.

*****The linear predicted value squared is not significant, indicating no evidence of misspecification.***

Step 3.

```
. logistic fraility_dich SI Age BMI i.Fracture i.SES  i.Alcohol i.Smoking i.CharlsonComorbidity
Fatmassindex xbhsq_SI xbhcu_SI, or base


. test xbhsq_SI xbhcu_SI

 ( 1)  [fraility_dich]xbhsq_SI = 0
 ( 2)  [fraility_dich]xbhcu_SI = 0

           chi2(  2) =    2.61
         Prob > chi2 =    0.2710
```

(Non-significant, no evidence of misspecification)

Exposure: HGS

Step 1.

```
. logistic fraility_dich HGS_std Age BMI i.Fracture i.SES  i.Alcohol i.Smoking i.CharlsonComorbidity
Fatmassindex, or base

Logistic regression                                     Number of obs =    344
                                                        LR chi2(14)   =  85.05
                                                        Prob > chi2   = 0.0000
Log likelihood = -106.98399                             Pseudo R2     = 0.2844

--------------------------------------------------------------------------------
  fraility_dich | Odds ratio   Std. err.      z    P>|z|     [95% conf. interval]
----------------+---------------------------------------------------------------
        HGS_std |      .1204    .041146    -6.19   0.000     .0616217    .2352445
            Age |   1.009655   .0213531     0.45   0.650     .9686593    1.052386
            BMI |   1.036798   .0741246     0.51   0.613     .9012365    1.192751
                |
       Fracture |
            0   |          1  (base)
            1   |   1.740556   .7764358     1.24   0.214      .726072    4.172498
                |
            SES |
            1   |          1  (base)
            2   |   .7858692   .3650619    -0.52   0.604     .3161836    1.953265
            3   |   .4020963   .1699814    -2.16   0.031      .175588       .9208
                |
        Alcohol |
            0   |          1  (base)
            1   |   1.072841   .5483383     0.14   0.891     .3939844    2.921406
                |
        Smoking |
            0   |          1  (base)
            1   |   .9275245   .7357721    -0.09   0.924     .1959285    4.390896
                |
```

```
CharlsonComor~y |
             0  |          1  (base)
             1  |    1.17331   .5410034     0.35   0.729     .4752599    2.896641
             2  |   1.004698   .5279366     0.01   0.993     .3587209    2.813939
             3  |   1.155243   .8552937     0.19   0.845     .2706943    4.930233
             4  |   2.956017   2.784631     1.15   0.250     .4665055    18.73083
             5  |   .5043338   .6802902    -0.51   0.612     .0358544     7.09405
             6  |          1  (empty)
             8  |          1  (empty)
                |
   Fatmassindex |   1.116072   .1062991     1.15   0.249     .9260201    1.345129
          _cons |   .0006583   .0014785    -3.26   0.001     8.07e-06    .0537112
---------------------------------------------------------------------------------
Note: _cons estimates baseline odds.
```

Step 2.

```
. linktest, nolog

Logistic regression                                     Number of obs =    344
                                                        LR chi2(2)    =  85.44
                                                        Prob > chi2   = 0.0000
Log likelihood = -106.79109                             Pseudo R2     = 0.2857

------------------------------------------------------------------------------
frailty_dich | Coefficient  Std. err.      z    P>|z|     [95% conf. interval]
-------------+----------------------------------------------------------------
       *_hat |   .8574051   .2671087     3.21   0.001     .3338817    1.380929
    **_hatsq |  -.0534168   .0887053    -0.60   0.547    -.2272759    .1204423
       _cons |   -.013089   .2298274    -0.06   0.955    -.4635424    .4373645
------------------------------------------------------------------------------
```

The linear predicted value has a low p-value indicating an acceptable predictability.
*****The linear predicted value squared is not significant, indicating no evidence of misspecification.***

Step 3.

```
. logistic fraility_dich HGS_std Age BMI i.Fracture i.SES i.Smoking i.CharlsonComorbidity Fatmassindex
xbhsq_HGS xbhcu_HGS, or base

. test xbhsq_HGS xbhcu_HGS

 ( 1)  [fraility_dich]xbhsq_HGS = 0
 ( 2)  [fraility_dich]xbhcu_HGS = 0

           chi2(  2) =    2.73
         Prob > chi2 =    0.2553
```

(Non-significant, no evidence of misspecification)

Women Data

Exposure: ALMI

Step 1.

```
. logistic fraility_dich rALM Age BMI i.Fracture i.SES i.Smoking i.CharlsonComorbidity Fatmassindex, or
base

Logistic regression                                     Number of obs =    311
                                                        LR chi2(13)   =  44.44
                                                        Prob > chi2   = 0.0000
Log likelihood = -109.84726                             Pseudo R2     = 0.1683

--------------------------------------------------------------------------------
  fraility_dich | Odds ratio   Std. err.      z    P>|z|     [95% conf. interval]
----------------+---------------------------------------------------------------
           ALMI |   .0032756   .0088914    -2.11   0.035      .000016    .6696394
            Age |   1.126256   .0282492     4.74   0.000     1.072227    1.183007
            BMI |   1.195011   .2576796     0.83   0.409     .7831187    1.823543
                |
       Fracture |
             0  |          1  (base)
             1  |    .951251   .3793142    -0.13   0.900     .4353875    2.078329
                |
            SES |
             1  |          1  (base)
             2  |   1.112307   .4792872     0.25   0.805     .4780207    2.588231
             3  |   1.180736   .5723954     0.34   0.732     .4565716    3.053494
                |
        Smoking |
             0  |          1  (base)
             1  |   1.799923   1.299834     0.81   0.416     .4370688    7.412388
                |
CharlsonComor~y |
             0  |          1  (base)
```

1	.9460996	.439805	-0.12	0.905	.3804051	2.35303
2	1.116951	.5642338	0.22	0.827	.4149969	3.006237
3	2.273709	1.70004	1.10	0.272	.5251656	9.844044
4	3.39875	4.381638	0.95	0.343	.2716175	42.52856
5	5.283059	7.930614	1.11	0.268	.2786841	100.1518
Fatmassindex	.9619163	.224255	-0.17	0.868	.609107	1.519081
_cons	3.53e-07	1.29e-06	-4.07	0.000	2.78e-10	.0004478

Note: _cons estimates baseline odds.

Step 2.

```
. linktest, nolog

Logistic regression                                     Number of obs =    311
                                                        LR chi2(2)    =  44.45
                                                        Prob > chi2   = 0.0000
Log likelihood = -109.84515                             Pseudo R2     = 0.1683

------------------------------------------------------------------------------
frailty_dich | Coefficient  Std. err.      z    P>|z|     [95% conf. interval]
-------------+----------------------------------------------------------------
       *_hat |   1.023776   .4031116     2.54   0.011     .2336915     1.81386
    **_hatsq |   .0082841   .1274166     0.07   0.948     -.241448    .2580161
       _cons |   .0081902   .3187208     0.03   0.979    -.6164911    .6328715
------------------------------------------------------------------------------
```

***The linear predicted value has a low p-value indicating an acceptable predictability.**
****The linear predicted value squared is not significant, indicating no evidence of misspecification.**

Step 3.

```
. logistic frailty_dich rALM Age BMI i.Fracture i.SES i.Smoking i.CharlsonComorbidity Fatmassindex
xbhsq_rALM xbhcu_rALM, or base

. test xbhsq_rALM xbhcu_rALM

 ( 1)  [frailty_dich]xbhsq_rALM = 0
 ( 2)  [frailty_dich]xbhcu_rALM = 0

           chi2(  2) =    1.67
         Prob > chi2 =    0.4337
```

(Non-significant, no evidence of misspecification)

Exposure: HGS

Step 1.

```
logistic fraility_dich SDunitsHGS Age BMI Height i.Fracture i.SES i.Smoking i.CharlsonComorbidity
Fatmassindex, or base

Logistic regression                                     Number of obs =    319
                                                        LR chi2(14)   =  61.16
                                                        Prob > chi2   = 0.0000
Log likelihood = -107.93872                             Pseudo R2     = 0.2208

--------------------------------------------------------------------------------
  fraility_dich | Odds ratio   Std. err.      z    P>|z|     [95% conf. interval]
----------------+---------------------------------------------------------------
     SDunitsHGS |   .4854853   .0760159    -4.62   0.000     .3571885    .6598644
            Age |   1.071296   .0270878     2.72   0.006     1.019499    1.125724
            BMI |   1.075025   .0436194     1.78   0.075     .9928432    1.164008
         Height |    .966749   .0305275    -1.07   0.284     .9087301    1.028472
                |
       Fracture |
             0  |          1  (base)
             1  |   .9696297   .3929142    -0.08   0.939     .4382089     2.14551
                |
            SES |
             1  |          1  (base)
             2  |   .8676334   .3738528    -0.33   0.742     .3728755    2.018872
             3  |   .9938547   .4908102    -0.01   0.990     .3775385    2.616282
                |
        Smoking |
             0  |          1  (base)
             1  |   1.180606   .8879975     0.22   0.825     .2703153    5.156316
                |
CharlsonComor~y |
             0  |          1  (base)
```

1	.8316095	.3878905	-0.40	0.693	.3333424	2.074667
2	.9029777	.4660296	-0.20	0.843	.3283744	2.483046
3	3.060282	2.317286	1.48	0.140	.6937778	13.49903
4	3.816273	5.798852	0.88	0.378	.1941928	74.99734
5	2.635381	3.903227	0.65	0.513	.1445906	48.03376
Fatmassindex	.9835912	.0468997	-0.35	0.729	.8958341	1.079945
_cons	.3646237	2.179019	-0.17	0.866	2.99e-06	44533.81

Note: _cons estimates baseline odds.

Step 2.

```
. linktest, nolog

Logistic regression                                     Number of obs =    274
                                                        LR chi2(2)    =  35.46
                                                        Prob > chi2   = 0.0000
Log likelihood = -97.920926                             Pseudo R2     = 0.1533

------------------------------------------------------------------------------
frailty_dich | Coefficient  Std. err.      z    P>|z|     [95% conf. interval]
-------------+----------------------------------------------------------------
      *_hat |   .7894532    .388664     2.03   0.042     .0276858    1.551221
    **_hatsq | -.0785075   .1320186    -0.59   0.552    -.3372592    .1802442
       _cons |  -.0685285    .341574    -0.20   0.841    -.7380013    .6009442
------------------------------------------------------------------------------
```

***The linear predicted value has a low p-value indicating an acceptable predictability.**
*****The linear predicted value squared is not significant, indicating no evidence of misspecification.**

Step 3.

```
. logistic fraility_dich SDunitsHGS Age BMI Height i.Fracture i.SES i.Smoking i.CharlsonComorbidity
Fatmassindex xbhsq_HGS xbhcu_HGS, or base

. test xbhsq_HGS xbhcu_HGS

 ( 1)  [fraility_dich]xbhsq_HGS = 0
 ( 2)  [fraility_dich]xbhcu_HGS = 0

           chi2( 2) =    0.69
         Prob > chi2 =    0.7097
```

(Non-significant, no evidence of misspecification)
